# Temporal Exercise Conditioning Confers Dual-Phase Cardioprotection Against Isoproterenol-Induced Injury in a Rat Model

**DOI:** 10.3390/antiox15020152

**Published:** 2026-01-23

**Authors:** Krisztina Kupai, Zsolt Murlasits, Hsu Lin Kang, Eszter Regős, Ákos Várkonyi, Csaba Lengyel, Imre Pávó, Zsolt Radák, Béla Juhász, Dániel Priksz, Anikó Pósa

**Affiliations:** 1 Department of Oral Biology and Experimental Dental Research, Faculty of Dentistry, University of Szeged, 6703 Szeged, Hungary; kupai.krisztina@med.u-szeged.hu (K.K.); hsu.lin-kang@o365.u-szeged.hu (H.L.K.);; 2 Department of Internal Medicine, Albert Szent-Györgyi Medical School, University of Szeged, 6703 Szeged, Hungary; 3 Institute of Sport Science and Physical Education, Faculty of Sciences, The University of Pecs, 7624 Pecs, Hungary; 4 Institute for Sports and Health Sciences, Hungarian University of Sports Science, 1123 Budapest, Hungary; 5 Department of Pharmacology and Pharmacotherapy, Faculty of Medicine, University of Debrecen, 4032 Debrecen, Hungarypriksz.daniel@pharm.unideb.hu (D.P.)

**Keywords:** cardioprotection, moderate exercise, preconditioning, postconditioning

## Abstract

Exercise training has demonstrated potential benefits in addressing the adverse effects of cardiovascular diseases, particularly myocardial infarction (MI). This study analyzed the cardioprotective effects of moderate exercise before and after MI in rats subjected to isoproterenol (ISO)-induced heart damage. *Wistar* rats were assigned to five groups: controls (CTRL), isoproterenol-treated (ISO), swimming before ISO (PRE + ISO), swimming after ISO (ISO + POST), and swimming both before and after ISO (PRE + ISO + POST). Cardiac function was assessed through echocardiography, while oxidative stress markers, Heme Oxygenase-1 (HO-1) and Myeloperoxidase (MPO), were quantified using biochemical assays and enzyme-linked immunosorbent assay (ELISA). Statistical analyses were conducted by one-way analysis of variance (ANOVA), accompanied by Tukey’s post hoc test. Exercise performed post-MI and both pre- and post-MI significantly reduced ISO-induced infarct size and improved left ventricular function (stroke volume (SV), ejection fraction (EF), and Tei index). HO-1 protein concentration and HO enzyme activity were restored, while swim training reduced the activity of MPO compared to the ISO group. Moderate exercise training, when appropriately timed, provides cardioprotection against ISO-induced myocardial damage by reducing oxidative stress and cardiac dysfunction.

## 1. Introduction

Cardiovascular diseases (CVDs) are a major global health concern, claiming millions of lives annually [1]. Among these, myocardial infarction (MI) is responsible for most deaths. Following an MI, the heart undergoes structural changes, especially in the left ventricle, which impairs left ventricular function. This pathological remodeling process involves an initial inflammatory response and subsequent scarring at the infarction site, along with alterations in the unaffected myocardium, such as interstitial fibrosis [2]. After MI, an inflammatory cascade is activated [3], which includes the secretion of inflammatory cells (e.g., macrophages, neutrophils) and the release of cytokines, such as Interleukin 6 (IL-6) and Tumor Necrosis Factor alpha (TNF-α) [4,5]. Ultimately, this remodeling can lead to a decline of ventricular function and an increased risk of death from cardiovascular causes [6].

However, convincing pre-clinical and clinical evidence indicates that exercise training can positively influence this remodeling process and enhance heart function after a MI [7,8,9,10,11,12]. In general, endurance exercise has been shown to protect against MI, with the magnitude of effect depending on the combination of training variables, such as exercise intensity, duration, and frequency. Through the production of protective proteins and enzymes, exercise induces a myriad of physiological responses that collectively lead to enhanced structural and functional integrity of the myocardium and a cardioprotective phenotype [13]. Recent meta-analytic evidence further strengthens these insights: exercise-based cardiac rehabilitation significantly improves left ventricular ejection fraction (LVEF) and early diastolic mitral inflow velocity (E) in post-MI patients, although other functional parameters may not show uniform benefits [14]. Another trial emphasized that early initiation of rehabilitation—particularly within the first two weeks post-MI—produces substantial improvements in exercise tolerance, including gains in metabolic equivalents (METs), 6 min walk distance, and heart rate recovery [15].

Inflammation is one of the most significant factors in the development of heart damage, and by reducing inflammatory markers, exercise training assists in the prevention of pathological remodeling and slowing of disease progression. Alemasi et al. [16] demonstrated that isoproterenol (ISO)-induced damage upregulated the gene expression of 20 cytokines in mice, of which 18 were counteracted by running exercise.

Furthermore, exercise can boost the body’s antioxidant defenses, which protects against oxidative stress, another contributor to CVDs [6,17,18]. A significant, exercise-induced cardioprotective molecule which has been associated with antioxidative, anti-inflammatory and anti-apoptotic effects is HO-1 [19]. In fact, Eltobshy et al. [20] reported that HO-1 upregulation led to improvements in cardiac morphology, electrical activity, and increased expression of endogenous antioxidants in an ISO-induced myocardial infarction model. In another study, while ISO administration increased total oxidant status, high-intensity interval training successfully restored total antioxidant capacity [12].

Although the benefits of exercise preconditioning prior to MI are widely established [5,8,12,13,21,22,23], the outcomes of post-MI exercise interventions are more controversial [24,25,26,27,28]. These post-MI studies demonstrated that the extent of myocardial damage could worsen, improve, or remain unchanged, depending on the elapsed time between the initial infarction and the initiation of the exercise intervention. Azamian Jazi et al. [24] implemented a 4-week exercise training program, which was initiated two days after MI and found that tissue injury was exacerbated compared to the control group. Other studies have shown significant improvements in cardiac remodeling and ventricular function following five weeks of exercise when the intervention started seven days post-MI [27,28]. Interestingly, this period corresponds to the time course of inflammation; thus, exercise seems to worsen the outcome during the early healing phase, while the same intervention can be beneficial when cardiac fibrosis is already apparent.

Understanding these mechanisms is essential for creating targeted exercise programs that can counteract pathological cardiac remodeling and maximize cardioprotection. As research progresses, it is likely that additional benefits of exercise on cardiovascular health will be uncovered, further emphasizing the importance of physical activity as a fundamental aspect of preventive and reconstructive medicine.

Therefore, in this investigation we aimed to analyze the potential cardioprotective effects of moderate exercise pre- and post-MI in rats subjected to ISO-induced heart damage. ISO is a non-selective α-adrenergic agonist which is widely used to induce myocardial damage via free radical production. The effects of ISO include damage, cell death, heart cell apoptosis, and depletion of energy in the heart cells, culminating in impaired cardiac function [29]. Importantly, these various cellular responses are similar to those seen in spontaneous and experimentally induced infarctions [30].

We hypothesized that exercise training would attenuate myocardial functional decline and protect the heart by suppressing oxidative signaling pathways and by stimulating the expression of protective molecules, such as HO-1.

## 2. Materials and Methods

### 2.1. Animals and Exercise Training

All the experiments were reviewed and approved by the Animal Welfare Committee of the University of Debrecen on 14 February 2024 (DEMAB/110-8/2023). In this study, male *Wistar* rats (BW 270–300 g) were used (BRC, Szeged, Hungary). Animals were kept at a standard 20–23 °C temperature, an appropriate light/dark cycle was provided, and husbandry conditions were accomplished according to Directive 2010/63/EU.

In our study, a maximum of 10 rats per group were used. At the beginning of the study, the rats were divided into five groups as follows: (1) non-interventional controls (CTRL), (2) isoproterenol-treated (ISO), (3) pre-treatment swimming training + ISO (PRE + ISO), (4) ISO + post-treatment swimming training (ISO + POST), and (5) pre-treatment swimming training + ISO + post-treatment swimming training (PRE + ISO + POST). Myocardial damage was induced by ISO injection; 1.0 mg/kg ISO (Sigma Chemicals Co., Ltd., Poole, UK) diluted in 1 mL of physiological saline was injected subcutaneously. In prior research, we established the appropriate ISO dosage and verified that it is sufficient to induce myocardial damage [4]. During the study, all efforts were made to minimize the stress for the animals.

**Figure 1:**
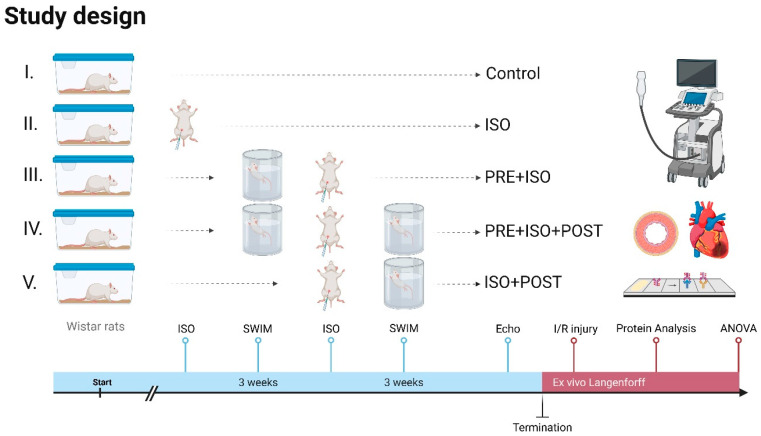
Study design. Rats received 3 weeks of forced swim training before and/or after isoproterenol-induced myocardial injury. High-resolution echocardiography was carried out at the endpoint, and ex vivo LAD-ligation was performed on a Langendorff apparatus to evaluate infarct size. Protein assays were carried out on myocardial samples to assess injury-induced changes. Created in Biorender. Dániel Priksz (2025) https://BioRender.com.

The animals were adapted to water one week before starting swimming training. The rats were then trained 5 days a week for 3 weeks individually in a 20 cm × 20 cm pool filled with >60 cm deep water at 33–34 °C to limit unwanted swimming behavior, such as bobbing, and to maintain constant limb movement [31]. Each training session lasted 40 min and was conducted in the mornings. The training sessions were monitored for continuous swimming without floating, thereby ensuring a moderate exercise intensity [31]. After swimming, the rats were dried with towels and returned to their cages. In the PRE + ISO group, animals trained for 3 weeks before ISO administration. The ISO + POST group received ISO injections and then trained for 3 weeks post-administration, followed by rest. The PRE + ISO + POST group trained for 3 weeks both before and after ISO treatment; Figure 1.

### 2.2. Experimental Protocol

All rats were sacrificed, and the hearts were perfused to wash out the remaining blood. The left ventricles were then harvested for biological measurements: HO activity, HO-1 concentration, and MPO activity. The left ventricular samples were powdered under liquid nitrogen and stored at −80 °C until the analyses.

#### 2.2.1. Protein Analysis

Using a commercial protein assay kit (Bio-Rad Labs, Hercules, CA, USA), 20 μL aliquots of the diluted samples were mixed with 980 μL of distilled water. Subsequently, 200 μL Bradford reagent was added to each sample. After mixing and incubating for 10 min, the samples were assayed spectrophotometrically at 595 nm. The protein level was expressed as mg protein/mL.

#### 2.2.2. Determination of Cardiac HO-1 Concentrations

A powdered ventricular sample of approximately 40 mg was homogenized in ice-cold phosphate buffer (pH 7.4) for 20 s. Supernatants were obtained after centrifugation at 4 °C (20 min, 2500 rpm) for ELISA and protein analyses. 40 μL supernatant of cardiac tissue or 50 μL standard solution was added into each well together with the monoclonal antibody following the manufacturer’s instructions (Gen Asia Biotech Co., Ltd., Shanghai, China). Next, we added 10 μL of the secondary antibody labeled with biotin to each well, followed by 50 μL Streptavidin-HRP to the supernatant and the standard solution, forming an immune complex with a biotin-labeled antibody. After 60 min incubation at 37 °C, the plate was washed 5 times to deplete unbound enzymes. Then, 50 μL of substrates A and B were added into the wells and incubated for 10 min at 37 °C for color development. At the final step, 50 μL of stop solution was added into wells and pipetted, resulting in color change from blue to yellow. Data was collected at 450 nm optical density using a Microplate reader (Bio-Rad, Hercules, CA, USA).

#### 2.2.3. Determination of Cardiac MPO Activity

Approximately 40 mg powdered ventricular tissue was homogenized in phosphate buffer (pH 6.0) containing 0.5% hexadecyltrimethylammonium bromide. The samples were first placed in liquid nitrogen and then transferred to a 37 °C water bath. This process was repeated three times. Next, samples were centrifuged (15,000× *g* for 15 min at 4 °C) and the supernatants were collected. On a 96-well plate, we added 280 μL (o-dianisidinediHCL along with 12 μL of sample or standard (diluted from peroxidase)) into the wells. Following agitation for 59 s, MPO activity was measured at 490 nm and expressed as μU/mg protein.

#### 2.2.4. Determination of Cardiac HO Activity

Cardiac tissue samples were homogenized in an ice-cold buffer containing 10 mM HEPES, 32 mM sucrose, 1 mM DTT, 0.1 mM EDTA, 10 μg/mL trypsin inhibitor, 10 μg/mL leupeptin, and 2 μg/mL aprotinin, pH 7.4. After centrifugation at 15,000 *g* for 20 min at 4 °C, the supernatant was discarded. The reaction mixture included 150.0 μL supernatant, 2.0 mM glucose-6-phosphate, 0.14 U/mL glucose-6-phosphate dehydrogenase, 15.0 μM hemin, 120.0 μg/mL rat liver cytosol (as a biliverdin reductase source), 2.0 mM MgCl_2__6H_2_O, and 100.0 mM KH_2_PO_4_. The reaction was initiated with 100.0 μL of reduced β-NADPH and subsequently incubated in the dark for 60 min at 37 °C. The bilirubin content was quantified by measuring the optical density at 465 nm and 530 nm and calculating the difference between the two measurements. HO activity was quantified as the amount of bilirubin (in nmol) produced per hour per milligram of protein.

#### 2.2.5. Echocardiography

Transthoracic echocardiography was performed under ketamine–xylazine anesthesia (70/5 mg/kg, i.m.). Data was collected in accordance with the American Society of Echocardiography recommendations [32,33]. A Vivid E9 sonograph (GE Healthcare, New York, NY, USA) with a linear i13L probe was employed, and the dataset was collected from the parasternal long and short axes, as well as the apical 4 chamber views. Ejection fraction (EF), fractional shortening (FS), and myocardial wall thickness during systole and diastole, as well as left atrial (LA) size, aortic (Ao) diameter, and mitral and tricuspid annular plane systolic excursion (MAPSE and TAPSE, respectively), were assessed using M-mode echocardiographic recordings. Additionally, pulsed wave (PW) Doppler and tissue Doppler imaging (TDI) echocardiography were performed to measure transmitral E and A wave velocities, the E/A ratio, and the deceleration time of the E wave (DecT). Wall motion was characterized by TDI-derived e’ and a’ waves, along with the corresponding e’/a’ ratios obtained at the mitral and septal annuli. The E/e’ ratio was subsequently calculated. Furthermore, ejection time (ET), isovolumic contraction time (IVCT), and isovolumic relaxation time (IVRT) were determined with the Tei index—also known as the myocardial performance index (MPI)—computed as the sum of IVRT and IVCT divided by ET. Left ventricular outflow tract (LVOT) velocities, both maximal and mean (LVOT V), as well as pressure gradients (LVOT PG), were recorded. Finally, heart rate (HR), stroke volume (SV), cardiac output (CO), and left ventricular mass were calculated.

#### 2.2.6. Ischemia/Reperfusion Protocol

Following anesthesia, heart tissues were rapidly excised and placed in ice-cold Krebs–Henseleit buffer solution consisting of 11.2 mM glucose, 1.24 mM KH_2_PO_4_, 20.1 mM NaHCO_3_, 119 mM NaCl, 4.7 mM KCl, 1.25 mM CaCl_2_, and 1.24 mM MgSO_4_ and then mounted onto a Langendorff perfusion system. Hearts were retrogradely perfused via the aorta at a constant pressure of 75 mmHg with the Krebs–Henseleit buffer which was bubbled with 5% CO_2_ and 95% O_2_ at 37 °C. After perfusion, local ischemia was induced by a 30 min occlusion of the left anterior descending coronary artery (LAD), followed by a reperfusion for 120 min. Upon the conclusion of each experiment, the LAD was reoccluded and perfusion stopped, and the hearts were stained using 1% Evans blue solution injected into the aorta to reveal the area at risk. Heart samples were then frozen at −20 °C overnight.

#### 2.2.7. Measurement of Infarct Size

Frozen heart-tissue samples were sliced into 2 mm thick sections and immersed in 1% 2,3,5-triphenyltetrazolium chloride (TTC) solution prepared in phosphate-buffered saline (pH 7.4) for 10 min at 37 °C. Following TTC staining, the tissue slices were transferred into 10% formalin solution for 10 min and then placed in phosphate buffer (pH 7.4). After incubation, each side of every slice was photographed with a digital camera. Infarct size was calculated as a percentage of the area at risk.

### 2.3. Statistical Analysis

Data are presented as the mean value of the group ± standard deviation (SD). To compare endpoint parameters—encompassing in vivo and in vitro data—across three groups, the Gaussian distribution was assessed using the Shapiro–Wilk normality test. Subsequently, statistical analyses were conducted employing one-way analysis of variance (ANOVA), accompanied by Tukey’s post hoc test, contingent upon the conditions that F achieved *p* < 0.05, the normality test was passed, and no significant heterogeneity of variance was detected. In instances where the normality test did not meet the required criteria, the Kruskal–Wallis test was utilized, followed by Dunn’s post hoc test. All statistical analyses were performed using GraphPad Prism software for Windows, version 9.00 (GraphPad Software Inc., La Jolla, CA, USA). Probability values (*p*) less than 0.05 were deemed statistically significant, and differences were denoted by asterisks (*).

## 3. Results

### 3.1. Infarct Size

**Figure 2:**
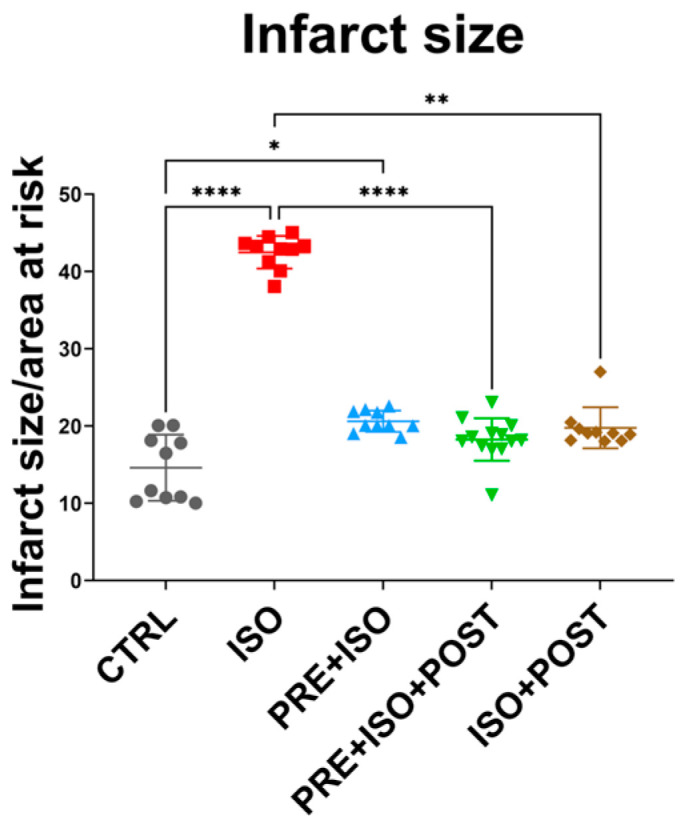
Infarct size after I/R injury among the treatment groups. Hearts isolated from rats in the different treatment arms were perfused via a Langendorff apparatus, and a 30 min ischemia was induced by the ligation of the LAD, which was followed by 120 min reperfusion. Hearts were then stained with TTC and the infarcted tissues were determined in 2 mm thick sections as a function or the total area at risk. The infarct size was significantly increased in the ISO group (red) compared to control, and was attenuated by the PRE + ISO + POST (green) and ISO + POST (brown) groups compared to ISO. **** *p* < 0.0001, ** *p* < 0.01, * *p* < 0.05.

As expected, infarct size significantly increased in the ISO-treated group compared to CTRL (from 14.61 ± 4.27% to 42.48 ± 2.11%; *p* < 0.001). Two of the training groups, PRE + ISO + POST and ISO + POST, showed a significant attenuation of infarct size compared to ISO (18.26 ± 2.75% and 19.77 ± 2.65%; *p* < 0.001 and *p* = 0.01, respectively). The attenuation in infarct size in the PRE + ISO group did not reach the level of significance (20.62 ± 1.38%; *p* = 0.22); however, there was a difference compared to the untreated CTRL group (*p* = 0.03; Figure 2).

### 3.2. Echocardiography

**Figure 3:**
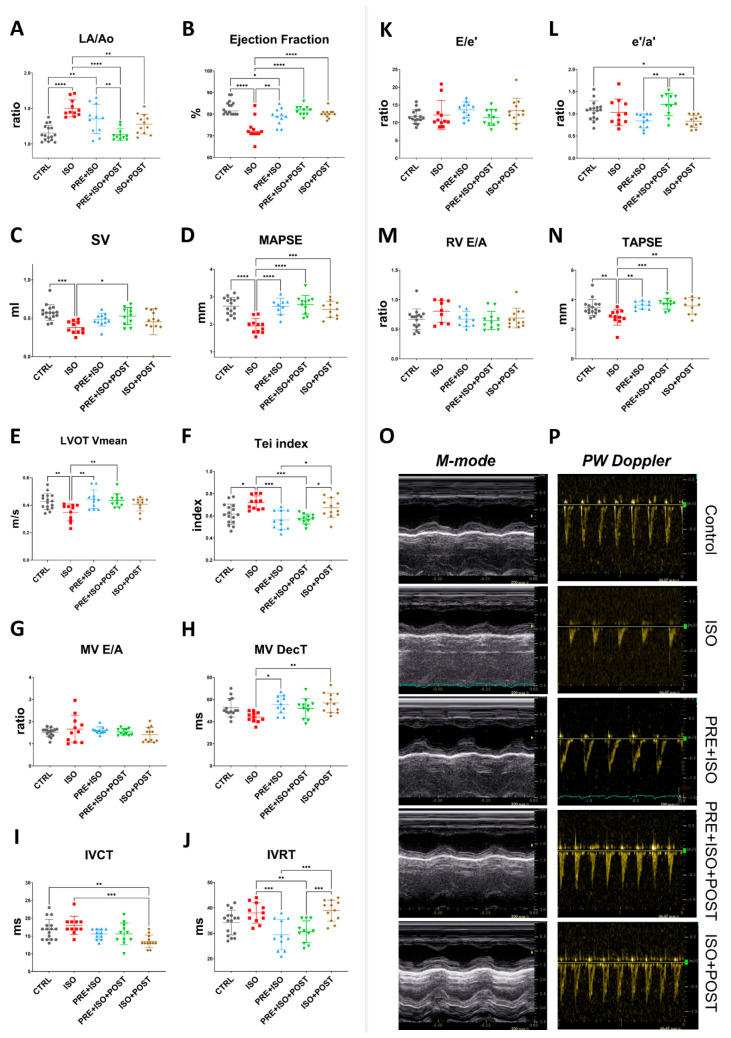
Endpoint echocardiographic parameters of treatment groups. (**A**) LA size increased in ISO but decreased in all treatment groups; PRE + ISO + POST treatment normalized LA size most effectively. (**B**) Ejection fraction was reduced in ISO and improved significantly in both PRE + ISO and PRE + ISO + POST groups. (**C**) Stroke volume decreased in ISO and was partially restored in PRE + ISO + POST, while PRE + ISO and ISO + POST showed lesser improvement. (**D**) MAPSE was diminished in ISO and fully normalized by PRE + ISO + POST; PRE + ISO and ISO + POST provided partial restoration. (**E**) LVOT mean velocity decreased in ISO, with PRE + ISO + POST and ISO + POST showing marked recovery compared to PRE + ISO. (**F**) Tei index increased in ISO and was significantly reduced in PRE + ISO + POST and ISO + POST, with PRE + ISO having a more pronounced effect. (**G**) MV E/A ratio was not significantly different among groups. (**H**) MV deceleration time slightly shortened in ISO, while PRE + ISO, PRE + ISO + POST, and ISO + POST all normalized DecT, with PRE + ISO + POST demonstrating the greatest effect. (**I**) IVCT was prolonged in ISO and improved in PRE + ISO + POST, with partial improvement in the PRE + ISO and ISO + POST groups. (**J**) IVRT was significantly lengthened after ISO and normalized with PRE + ISO + POST and PRE + ISO, but not in ISO + POST. (**K**) E/e’ ratio was unchanged among groups. (**L**) The e’/a’ ratio slightly decreased in ISO, but was normal in PRE + ISO + POST. (**M**) RV E/A ratio did not show any significant differences among groups. (**N**) TAPSE was significantly decreased in ISO and largely restored by PRE + ISO + POST, with PRE + ISO and ISO + POST showing only partial improvement. (**O**) Representative M-mode image, sweep speed: 200 mm/s and (**P**) PW Doppler traces of treatment groups, sweep speed: 66.67 mm/s. LA: left atrium; Ao: aortic diameter; EF: ejection fraction; SV: stroke volume; MAPSE: mitral annular plane systolic excursion; LVOT Vmean: left ventricular outflow tract mean velocity; Tei index: myocardial performance index; MV E/A: mitral valve early-to-late diastolic inflow velocity ratio; MV DecT: mitral valve deceleration time; IVCT: isovolumetric contraction time; IVRT: isovolumetric relaxation time; E/e’: early diastolic mitral inflow velocity to early mitral annular velocity ratio; e’/a’: early-to-late diastolic mitral annular tissue velocity ratio; RV E/A: right ventricular early-to-late diastolic inflow velocity ratio; TAPSE: tricuspid annular plane systolic excursion. One-way ANOVA with Tukey’s post hoc test and Kruskal–Wallis test with Dunn’s post hoc test were used to evaluate differences among groups; *: *p* < 0.05; **: *p* < 0.01; ***: *p* < 0.001; ****: *p* < 0.0001.

The Left atrium/Aortic root ratio (La/Ao) increased, indicating structural changes in the ISO-treated hearts (from 1.16 ± 0.11 to 1.50 ± 0.12; *p* < 0.001 vs. CTRL, Figure 3A). The PRE + ISO + POST and ISO + POST training groups showed a significant attenuation in the left atrial size when compared to the ISO group (1.35 ± 0.21 and 1.28 ± 0.14; *p* < 0.001 and *p* = 0.00 vs. ISO, respectively). No significant changes in relative wall thickness (RWT) were observed in any experimental group at the study endpoint, regardless of isoproterenol treatment or exercise training. The Tei index, also known as the myocardial performance index (MPI), significantly worsened with ISO treatment compared to control (from 0.62 ± 0.09 to 0.72 ± 0.06; *p* = 0.01 vs. CTRL). Exercise pre- and both pre- and post-MI (PRE + ISO, PRE + ISO + POST) improved this measure (0.56 ± 0.09 and 0.57 ± 0.04; *p* = 0.00 and *p* = 0.00 vs. ISO, respectively), indicating an improvement in overall cardiac function (Figure 3F). As regards systolic function of the heart, ejection fraction (EF) significantly decreased in the ISO group compared to CTRL (from 82.88 ± 3.20% to 73.09 ± 5.03%; *p* < 0.001). When compared to ISO, exercise training improved EF in all groups, completely restoring it in the PRE + ISO + POST and ISO + POST groups (81.64 ± 2.16% and 80.25 ± 1.96%, *p* < 0.001 and *p* = 0.01 vs. ISO, respectively (Figure 3B). Stroke volume decreased significantly in ISO vs. control (0.57 ± 0.10 mL and 0.38 ± 0.08 mL, *p* = 0.00 vs. control). Interestingly, SV was restored only in the PRE + ISO + POST group (0.53 ± 0.11 mL, *p* = 0.02 vs. ISO; Figure 3C). Similarly, mitral annular plane systolic excursion (MAPSE), indicative of LV function, deteriorated in response to ISO treatment (from 2.66 ± 0.31 mm to 1.93 ± 0.26 mm; *p* < 0.001 vs. control). MAPSE recovered in all three exercise training groups and was not different from normal CTRL (2.65 ± 0.29 mm, 2.71 ± 0.34 mm, and 2.54 ± 0.30 mm in PRE + ISO, PRE + ISO + POST, and ISO + POST, with *p* < 0.001, *p* = 0.00, and *p* = 0.00 vs. ISO, respectively; Figure 3D). Right ventricle systolic capacity, measured as tricuspid annular plane systolic excursion (TAPSE), also worsened in the ISO group compared to healthy controls (from 3.47 ± 0.54 mm to 2.82 ± 0.54 mm; *p* = 0.01 vs. CTRL). TAPSE was found to be indifferent from control in all three treatment groups (3.63 ± 0.26 mm, 3.73 ± 0.37 mm, and 3.58 ± 0.56 mm in PRE + ISO, PRE + ISO + POST, and ISO + POST, with *p* = 0.00, *p* = 0.00, and *p* = 0.01 vs. ISO, respectively; Figure 3N). Mean left ventricular outflow tract pressure gradient (LVOT maxPG) reduced with ISO administration in comparison to control values (from 2.36 ± 0.61 mmHg to 1.28 ± 0.28 mmHg, *p* < 0.001 vs. CTRL). Irrespective of the timing of exercise training, LVOT max pressure returned to the control levels (2.40 ± 0.52 mmHg, 2.53 ± 0.36 mmHg, and 2.27 ± 0.42 mmHg in PRE + ISO, PRE + ISO + POST, and ISO + POST, with *p* = 0.00, *p* = 0.01, and *p* = 0.03 vs. ISO, respectively). LVOT mean velocity followed a similar pattern. LVOT Vmean was decreased in ISO vs. control (from 0.43 ± 0.06 m/s to 0.35 ± 0.06 m/s; *p* < 0.001 vs. CTRL), while it was restored to the control level in the PRE + ISO and PRE + ISO + POST groups (0.44 ± 0.07 m/s and 0.43 ± 0.05 m/s in PRE + ISO and PRE + ISO + POST, with *p* = 0.00 and *p* = 0.01 vs. ISO, respectively; Figure 3E). Interestingly, LVOT Vmean did not increase significantly in the ISO + POST group (0.41 ± 0.05 m/s, *p* = ns vs. ISO).

Prominent differences in diastolic heart function were observed amongst the study groups. While the E/A and E/e’ ratios were relatively unchanged due to high variations among the rats (Figure 3G,K), E wave deceleration time (DecT) shortened in the ISO group (from 52.57 ± 8.33 ms to 44.09 ± 4.63 ms, *p* = 0.075 vs. control). DecT was normalized in the PRE + ISO group (55.64 ± 7.93 ms) and in the PRE + ISO + POST group (51.91 ± 8.93 ms) compared to ISO (44.09 ± 4.63 ms, *p* = 0.01 and *p* = 0.00, respectively, Figure 3H). The ratio of tissue e’/a’ was the highest in the PRE + ISO + POST group (1.21 ± 0.25) and differed significantly from PRE + ISO (0.84 ± 0.16, *p* = 0.00) and ISO + POST (0.83 ± 0.14, *p* = 0.00; Figure 3L). IVRT was lengthened in the ISO + POST group (38.92 ± 4.1 ms) compared to the PRE + ISO (29.45 ± 5.95, *p* = 0.00) and PRE + ISO + POST groups (30.64 ± 4.32 ms, *p* = 0.01). In these latter two groups, IVRT was significantly reduced compared to the ISO group (38.00 ± 4.00 ms, *p* = 0.00 and *p* = 0.01, respectively; Figure 3J). Flow propagation velocity (Vp) decreased in ISO (50.09 ± 20.37 cm/s) compared to control (71.44 ± 13.99 cm/s; *p* = 0.03 vs. ISO) and was unchanged in the treatment groups. Other determined echocardiographic parameters of treatment groups are listed in Supplementary Table S1.

### 3.3. HO-1 Concentration

**Figure 4:**
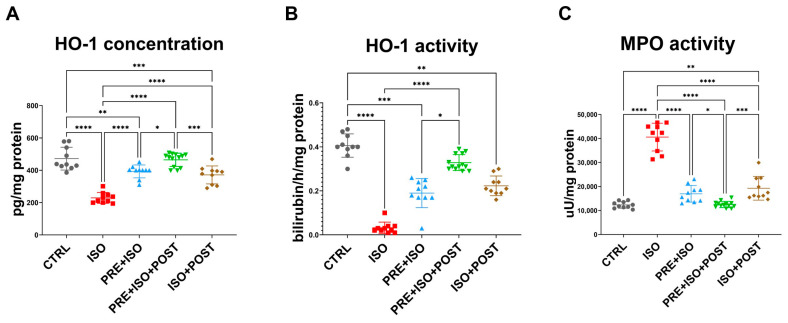
(**A**) Heme-oxigenase-1 (HO-1) concentration significantly decreased in myocardial samples of ISO-treated rats, while it was restored in all treatment groups, reaching the highest expression in the PRE + ISO +POST group (**B**) The enzyme activity of HO-1 dramatically decreased in the diseased group, but increased after the pre-and post-treatments, reaching the highest activity in the PRE + ISO + POST animals. (**C**) Myeloperoxidase (MPO) activity increased in ISO, but decreased in all treatment groups. One-way ANOVA with Tukey’s post hoc test was used to evaluate differences among groups; *: *p* < 0.05; **: *p* < 0.01; ***: *p* < 0.001; ****: *p* < 0.0001.

While HO-1 concentration decreased as a result of ISO administration (from 472 ± 70.83 pg/mg of protein to 229.3 ± 33.47 pg/mg of protein; *p* < 0.001 vs. control), in all training groups (PRE + ISO, PRE + ISO + POST, ISO + POST), HO-1 protein concentration was restored to baseline levels (393 ± 39.84 pg/mg of protein, 464.6 ± 41.26 pg/mg of protein, and 371.4 ± 55.55 pg/mg of protein; *p* < 0.001, *p* < 0.001, and *p* < 0.001 vs. ISO, respectively; Figure 4A).

### 3.4. HO Activity

HO activity decreased in the ISO-treated animals (from 0.406 ± 0.053 bilirubin/h/mg of protein to 0.032 ± 0.025 bilirubin/h/mg of protein; *p* < 0.0001 vs. CTRL), along with the protein concentration. When exercise training was administered both pre- and post-infarct (PRE + ISO and ISO + POST groups), HO activity returned to normal and was significantly higher than in ISO (0.19 ± 0.07 bilirubin/h/mg of protein and 0.22 ± 0.044 bilirubin/h/mg of protein; *p* < 0.0001 and *p* < 0.001 vs. ISO, respectively). The enzyme activity in the PRE + ISO + POST group was the closest to the normal level (0.33 ± 0.04 bilirubin/h/mg of protein; *p* = 0.00 vs. ISO; Figure 4B).

### 3.5. MPO Activity

ISO treatment led to a significant increase in MPO activity (from 12,342 ± 1387 µU/mg protein to 40,597 ± 5786 µU/mg protein; *p* < 0.001 vs. CTRL), which was attenuated by exercise and returned to baseline levels in all training groups, PRE + ISO, PRE + ISO + POST, and ISO + POST (16,967 ± 3453 µU/mg protein, 12,516 ± 1308 µU/mg protein, and 19,260 ± 4936 µU/mg protein; *p* < 0.001, *p* < 0.001 and *p* < 0.001 vs. ISO, respectively; Figure 4C).

## 4. Discussion

The current study demonstrated that moderate swim training provides significant cardioprotection in rats subjected to ISO treatment. Notably, this was the first investigation to systematically compare the effects pre- and post-MI exercise training in this model. Most preclinical work tests either preconditioning or postconditioning separately or compares exercise to classical ischemic preconditioning rather than analyzing the effects of exercise pre- and post-conditioning together, especially in the ISO model. A similar experimental study in ischemia–reperfusion and pharmacologic conditioning models demonstrates that dual-phase (pre + post) strategies can produce superior cardioprotection [34]. These strategies engage complementary mechanisms such as mitochondrial biogenesis, preservation of mitochondrial function, inhibition of inflammation, and activation of PI3K/Akt signaling [34]. Mitochondrial biogenesis enhances cellular energy production, while PI3K/Akt signaling promotes cell survival, both of which are crucial for reducing cardiac injury. This supports the novelty and mechanistic plausibility of our findings when moderate exercise is applied both before and after myocardial damage.

According to the collective echocardiographic and molecular data presented here, moderate exercise performed both pre- and post-MI was the most effective for attenuating ISO-induced myocardial damage. Our study also observed that exercise after myocardial infarction (MI) had a positive effect. This finding aligns with some studies, though it contradicts others [24,25,26,27,28].

The results suggest that only PRE + ISO + POST and ISO + POST exercise protocols resulted in a reduction in infarct size, while exercise prior to MI did not have a significant effect on myocardial damage. Considering the overwhelming scientific evidence on the role of exercise training in preconditioning, this finding was unexpected [5,12,23,27,35,36,37]. There may be a statistical reason for this outcome, as the values were similar to those observed in other exercise groups. However, the groups demonstrated an abnormal distribution. In fact, many important functional parameters, such as Tei index, EF, and MAPSE, improved in the PRE + ISO group compared to ISO, implying the effectiveness of prior exercise training in cardioprotection. (Figure 3). Nevertheless, Veiga et al. [38] also reported that 8 weeks of swim training prior to ischemia–reperfusion injury did not attenuate myocardial damage. However, there were important differences in the study design compared to our investigation. The researchers used female rats and performed coronary ligation, including only animals with moderate and large infarct sizes, which may have influenced the results. Furthermore, exercise preconditioning can improve ventricular performance through mechanisms that do not necessarily reduce infarct size [39]. Endurance exercise and preconditioning increase myocardial angiogenesis and capillary density via myokine and vascular endothelial growth factor (VEGF) signaling, improve microvascular perfusion of the peri-infarct border zone, and thereby preserve contractile function (EF, MAPSE, and Tei) without a proportional decrease in measured infarct area [40].

Temporal factors can also contribute to this outcome; infarct size assays quantify irreversible myocardial damage at a single timepoint, whereas echocardiographic indices reflect integrated ventricular performance that is rapidly influenced by reduced edema, less stunning, and improved microvascular flow [41].

Exercise training significantly mitigated ISO-induced cardiac disfunction (Figure 3). Although SV was improved only in the PRE + ISO + POST group, all groups exhibited a significant difference from ISO in EF and MAPSE. There was a significant reduction in left ventricular outflow tract pressure gradient in the ISO-treated animals, which was normalized in all treatment groups. Diastolic function showed less marked changes as E/A and E/e’ were unaffected by the treatments. However, when evaluating more heart rate-independent parameters, we observed a shortened E wave deceleration time (DecT) and increased isovolumic contraction time (IVRT) in the ISO group, and normalized DecT in the PRE + ISO and ISO + POST animals, as well as normal IVRT in the PRE + ISO and PRE + ISO + POST groups. Interestingly, IVRT was significantly lengthened in the ISO + POST group, showing the deteriorative effects of post-infarction exercise on diastolic function. Tei index, showing global cardiac function, deteriorated in the ISO group, but recovered in both the PRE + ISO and PRE + ISO + POST groups. These findings agree with previous data, demonstrating that exercise training is capable of restoring both systolic and diastolic functions [42,43,44]. While the measured parameters often differed from the current analyses, exercise training has consistently improved EF in ISO-induced myocardial damage, as observed in our study. Although we did not measure fibrotic markers in our study, it is well accepted that ISO administration leads to cardiac fibrosis, resulting in myocardial disfunction, and exercise training has been shown to counteract this negative effect [28,45,46].

The administration of ISO resulted in a marked increase in the inflammatory molecule MPO, while concurrently reducing the level and inhibiting the activity of the antioxidant enzyme HO-1/HO in the left ventricle. On the other hand, when moderate swim training preceded or followed ISO treatment, the levels of these molecules returned to control levels. HO-1 is a stress-induced protein that is essential in heme metabolism as it catalyzes the conversion of heme into free iron, carbon monoxide (CO), and biliverdin [47]. Biliverdin/bilirubin and CO possess anti-inflammatory, anti-apoptotic, and antioxidant properties. Therefore, restoring HO-1 protein expression may have contributed to the cardioprotection observed in our study.

In addition to enhancing the expressions and activity of the antioxidant and anti-inflammatory protein HO-1/HO, exercise training also inhibited the inflammatory molecule MPO. These results confirm our previous findings in estrogen-deficient female animals that exercise training was effective in reducing MPO and increasing HO-1 and GSH after ISO treatment [26]. MPO is a mammalian heme peroxidase and a principal pro-inflammatory and oxidative enzyme which plays a role in the inflammatory response following myocardial damage [48]. MPO also predicted coronary artery disease (CAD) and cardiovascular mortality risk in patients undergoing coronary angiography [49]. Interestingly, it has been demonstrated that antioxidant treatment can effectively mitigate increased MPO levels [50]. The marked reduction in MPO activity observed in this study underscores its role as a crucial mechanism of cardioprotection. This outcome not only highlights the anti-inflammatory benefits of exercise but also aligns with previous preclinical and clinical findings, demonstrating that controlling MPO activity can substantially decrease adverse cardiac remodeling and the risk of cardiovascular complications [48,51]. In fact, prolonged elevation of the enzyme is associated with long-term adverse events, including reduced cardiac function and survival rates [52]. The inhibition of MPO by exercise likely represents a key pathway through which cardioprotection is achieved, emphasizing the therapeutic potential of moderate exercise in counteracting ISO-induced cardiac injury.

This study has several limitations that should be acknowledged. First, the use of a rat model, while valuable for mechanistic insights, may limit the direct generalizability of the findings to human populations, as physiological responses to exercise and myocardial injury can differ across species. Second, although we assessed key molecular markers of oxidative stress and inflammation, we did not directly quantify reactive oxygen species (ROS), which could provide a more comprehensive understanding of the oxidative mechanisms involved. Third, there is inherent variability in exercise response among individual animals, which may influence the observed outcomes and should be considered when interpreting the results.

While our rat swimming protocol models key physiological responses to exercise pre- and post-conditioning, it is not directly comparable to human cardiopulmonary exercise testing (CPET) or clinical exercise interventions. Differences in species physiology, exercise modality (forced swimming versus treadmill/cycle or volitional exercise), and the integrated cardiorespiratory measurements obtainable in humans mean that our results should be interpreted as mechanistic rather than directly predictive of clinical outcomes. Nevertheless, the dual-phase cardioprotective patterns we observed suggest specific hypotheses that could be tested in humans using established CPET endpoints [53,54].

Previous research has indicated that isoproterenol induces myotoxic effects by increasing oxidative stress and inflammation [55,56]. Although we did not measure other antioxidant molecules in this investigation, Ma et al. [45] reported that swim training resulted in a significant increase in Superoxide Dismutase (SOD)1 and SOD2 mRNA and protein expressions in ISO-treated mice. To gain a comprehensive understanding of the mechanisms involved, future research should also include reactive oxygen species (ROS) measurement in this model. Such measurements would clarify whether the observed cardioprotective effects of exercise are primarily due to the suppression of oxidant production or an increase in ROS scavenging. This additional data could provide valuable insights into the specific pathways through which exercise exerts its beneficial effects, ultimately leading to more targeted therapeutic strategies.

## 5. Conclusions

These findings underscore the importance of exercise both before and after cardiotoxic events for providing the most comprehensive cardioprotection. The recovery of heart function and molecular markers was most pronounced in the PRE + ISO + POST group, restoring antioxidant HO-1 protein concentration and HO enzyme activity to baseline levels, and significantly attenuating inflammatory MPO activity. While exercise-induced preconditioning and postconditioning involve complex and overlapping mechanisms, it is feasible that some molecular pathways differ and contribute to resistance to myocardial damage [57]. Existing ischemia–reperfusion and exercise studies support distinct mechanisms for preconditioning (angiogenesis, mitochondrial biogenesis, antioxidant enzyme upregulation) and postconditioning (attenuation of reperfusion oxidative stress, anti-inflammatory signaling), providing a mechanistic rationale for additive benefit when both phases are combined [58,59]. As a result, when both sets of mechanisms are engaged in the PRE + ISO + POST group, the myocardium may benefit from both increased resistance to the insult and enhanced early repair, producing more complete functional and molecular recovery.

## Data Availability

The original contributions presented in this study are included in the article/Supplementary Material. Further inquiries can be directed to the corresponding author.

## Supplementary Materials

The following supporting information can be downloaded at https://www.mdpi.com/article/10.3390/antiox15020152/s1. Table S1: Endpoint echocardiographic parameters of treatment groups
