# Supplementary material for: Temporal Exercise Conditioning Confers Dual-Phase Cardioprotection Against Isoproterenol-Induced Injury in a Rat Model
Source: Antioxidants (Basel). 2026 Jan 23;15(2):152. doi: 10.3390/antiox15020152 (PMC12938271; doi:10.3390/antiox15020152)
Supplement: Supplementary file 1 [file antioxidants-15-00152-s001.zip › antioxidants-4032540-supplementary.pdf]

	CTRL	ISO	PRE+ISO	PRE+ISO+POST	ISO+POST
**LA/Ao ratio**	1.152±0.112	1.501±0.119	1.352±0.207	1.129±0.082	1.278±0.138
**EF (%)**	82.88±3.202	73.09±5.029	78.91±3.807	81.64±2.157	80.25±1.96
**MAPSE (mm)**	2.66±0.311	1.936±0.263	2.651±0.292	2.711±0.3417	2.54±0.297
**Vmax (m/s)**	0.761±0.095	0.558±0.063	0.77±0.0824	0.794±0.059	0.755±0.073
**maxPG (mmHg)**	2.355±0.613	1.276±0.276	2.395±0.519	2.525±0.363	2.269±0.418
**E/A ratio**	1.523±0.189	1.61±0.6176	1.615±0.148	1.53±0.145	1.408±0.331
**MV DecT (ms)**	52.57±8.336	44.09±4.636	55.64±7.928	51.91±8.927	56.83±8.799
**e'/a' ratio**	1.073±0.216	1.032±0.299	0.8405±0.159	1.21±0.252	0.831±0.136
**E/e' ratio**	11.77±1.954	12.19±4.053	13.62±2.263	11.44±2.241	13.37±3.54
**Tei index**	0.615±0.090	0.719±0.061	0.562±0.084	0.570±0.044	0.673±0.0892
**IVCT (ms)**	16.81±2.903	18±2.53	15.55±1.44	15.64±3.075	13.5±1.784
**IVRT(ms)**	34.25±4.837	38.0±4.0	29.45±5.956	30.64±4.319	38.92±4.1
**SV (mL)**	0.572±0.103	0.378±0.078	0.480±0.083	0.526±0.112	0.452±0.169
**RV E/A ratio**	0.669±0.176	0.803±0.187	0.671±0.128	0.647±0.155	0.69±0.1667
**TAPSE (mm)**	3.47±0.538	2.816±0.538	3.634±0.262	3.729±0.37	3.578±0.5581
**RWT (%)**	0.541±0.068	0.417±0.077	0.4902±0.109	0.5083±0.065	0.5424±0.086
**Vp (cm/s)**	71.44±13.99	50.09±20.37	63.22±9.311	61.9±19.81	66.44±10.97

Supplementary Table 1. Endpoint echocardiographic parameters of treatment groups. LA/Ao: Left atrium to aortic root diameter ratio; EF: Ejection fraction; MAPSE: Mitral annular plane systolic excursion; Vmax: Maximal velocity of blood flow through the left ventricular outflow tract,; maxPG: Maximal pressure gradient across the left ventricular outflow tract; E/A ratio: Ratio of early (E) to late (A) ventricular filling velocities; MV DecT: Mitral valve deceleration time; e'/a': Ratio of early (e') to late (a') diastolic mitral annulus tissue velocities; E/e': Ratio of early mitral inflow velocity to mitral annulus early diastolic velocity; Tei index: Myocardial performance index; IVCT: Isovolumic contraction time; IVRT: Isovolumic relaxation time,; SV: Stroke volume; RV E/A: Right ventricular early to late diastolic filling velocity ratio; TAPSE: Tricuspid annular plane systolic excursion; RWT: Relative wall thickness; Vp: Flow propagation velocity.
